# Tanycytic Ependymoma: A Challenging Histological Diagnosis

**DOI:** 10.1155/2013/170791

**Published:** 2013-02-14

**Authors:** Khaled M. Krisht, Meic H. Schmidt

**Affiliations:** Department of Neurosurgery, Clinical Neurosciences Center, University of Utah, Salt Lake City, UT 84132, USA

## Abstract

Tanycytic ependymoma is a rare form of ependymoma that usually arises in the intramedullary spine. It has a unique histology emphasized by the inconspicuous ependymal pattern of cells and close resemblance to schwannoma and astrocytoma. The authors report a 50-year-old man with a cervical tanycytic ependymoma that was initially thought to be a schwannoma. The frozen histology section showed spindle cells with oval and elongated nuclei with occasional hemosiderin deposits present suggesting a preliminary diagnosis of schwannoma. Immunohistochemical staining of the permanent section revealed strong immunoreactivity for glial fibrillary acidic protein with intermittent S-100 positivity, confirming that the tumor was a tanycytic ependymoma. This underlines the challenges involved in making an accurate diagnosis and demonstrates that careful and detailed histological inspection with immunohistochemical stains and ultrastructural microscopy may be necessary to distinguish tanycytic ependymoma from other neoplasms.

## 1. Introduction

 Tanycytic ependymoma is a World Health Organization (WHO) grade II tumor with histological characteristics distinct from the typical features of commonly encountered ependymomas. In these lesions, the classic ependymal rosettes and perivascular pseudorosettes are replaced by more fibrillar cells. Thus, the cellular appearance and arrangement may mimic other lesions such as schwannoma and astrocytoma, which makes the diagnosis challenging and hence warrants further evaluation. We present a rare case of an intramedullary spinal tumor in a 50-year-old man in whom the preliminary intraoperative diagnosis of schwannoma was revised to tanycytic ependymoma after the application of immunohistochemical stains and careful ultrastructural interpretation.

## 2. Case Presentation

### 2.1. History and Examination

A 50-year-old man with a five-year history of progressively worsening upper extremity numbness presented to an outside physician with the recent onset of bilateral lower extremity numbness and gait disturbance. He was referred to our neurosurgery clinic after magnetic resonance (MR) imaging of the cervical spine revealed an intramedullary lesion at the C3–5 level. His neurologic examination showed that he had diminished soft touch and pinprick sensation involving both shoulders, arms, and hands along the C5, C6, and C7 dermatomal distributions. The patient was noted to be hyperreflexive, with 3+ brachioradialis and patellar reflexes, as well as myelopathic, with evident Hoffman sign and clonus and a positive Romberg sign. There was no evidence of bladder, bowel, and/or sexual dysfunction.

**Figure 1:**
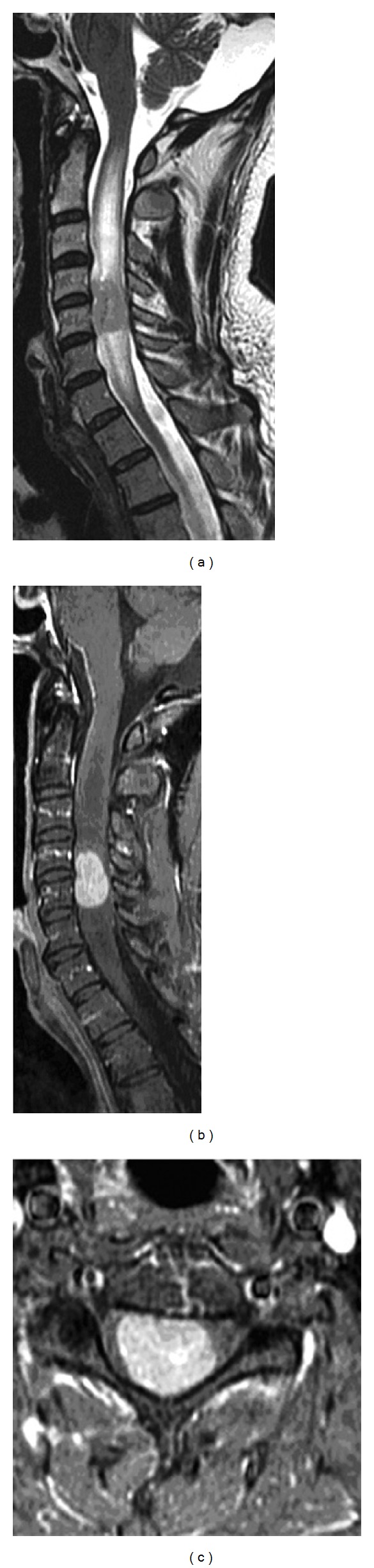
(a) Sagittal T2-weighted image of the cervical spine with an isointense lesion at C4-5 with surrounding T2 hyperintensity suggestive of edema. (b) Sagittal T1-weighted image with gadolinium enhancement showing an avidly enhancing lesion with surrounding cord edema. (c) Axial T1-weighted image with gadolinium enhancement revealing an intramedullary homogenously enhancing lesion with severe cord compression.

 On MR imaging, the tumor measured 2.4 × 1.9 × 1.3 cm, spanning C3 through C5. It was avidly enhancing on T1-weighted imaging, mostly centered at the C4 level, and hypointense on T2-weighted imaging, with apparent hyperintensity at the inferior and superior poles of the lesion suggestive of vasogenic edema (Figures 1(a)–1(c)).

### 2.2. Surgical Procedure and Outcome

**Figure 2:**
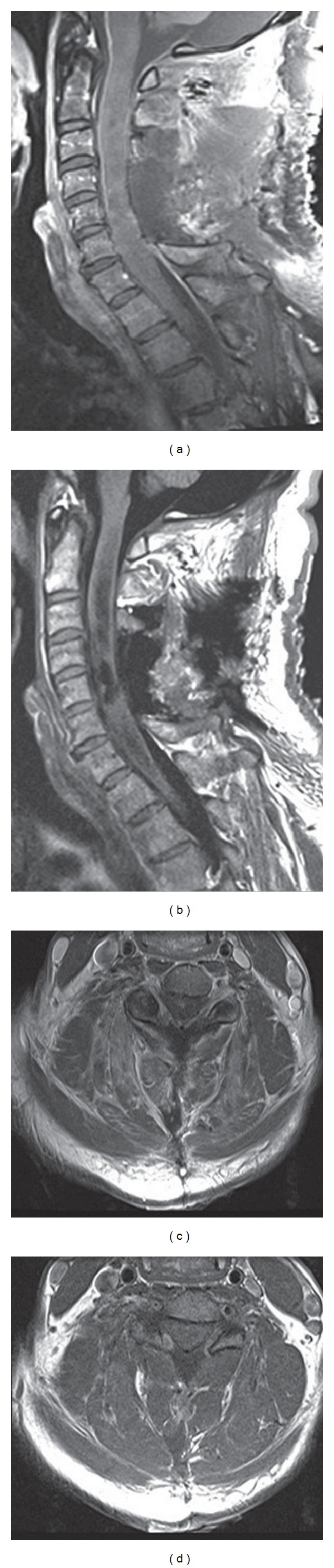
((a), (c)) Sagittal and axial gadolinium enhanced T1-weighted images of the cervical spine after C3–C6 laminectomies and tumor resection demonstrating GTR with no residual enhancement. ((b), (d)) Sagittal and axial T1-weighted inversion recovery images of the cervical spine after tumor resection demonstrating persistent cord syrinx and edema.

The tumor was exposed via a C3–6 laminectomy. The craniocaudal extent of the lesion was identified with intraoperative ultrasound before we proceeded with the durotomy. After the durotomy was completed and the dural leaflets were reflected, a midline myelotomy was performed. The tumor was noted to be purplish in color and moderately vascular. There appeared to be a clear plane between the tumor and spinal cord; however, because of the large size of the tumor, a Cavitron ultrasonic surgical aspirator was first used to debulk the tumor and decompress the cord. After the tumor size was reduced, it became easier to mobilize the remainder of the mass, which allowed for the careful peeling of the ventral capsule and achievement of gross total resection (GTR) (Figure 2). Intraoperative neuromonitoring with somatosensory-evoked potentials and motor-evoked potentials remained stable.

### 2.3. Histopathological Analysis

**Figure 3:**
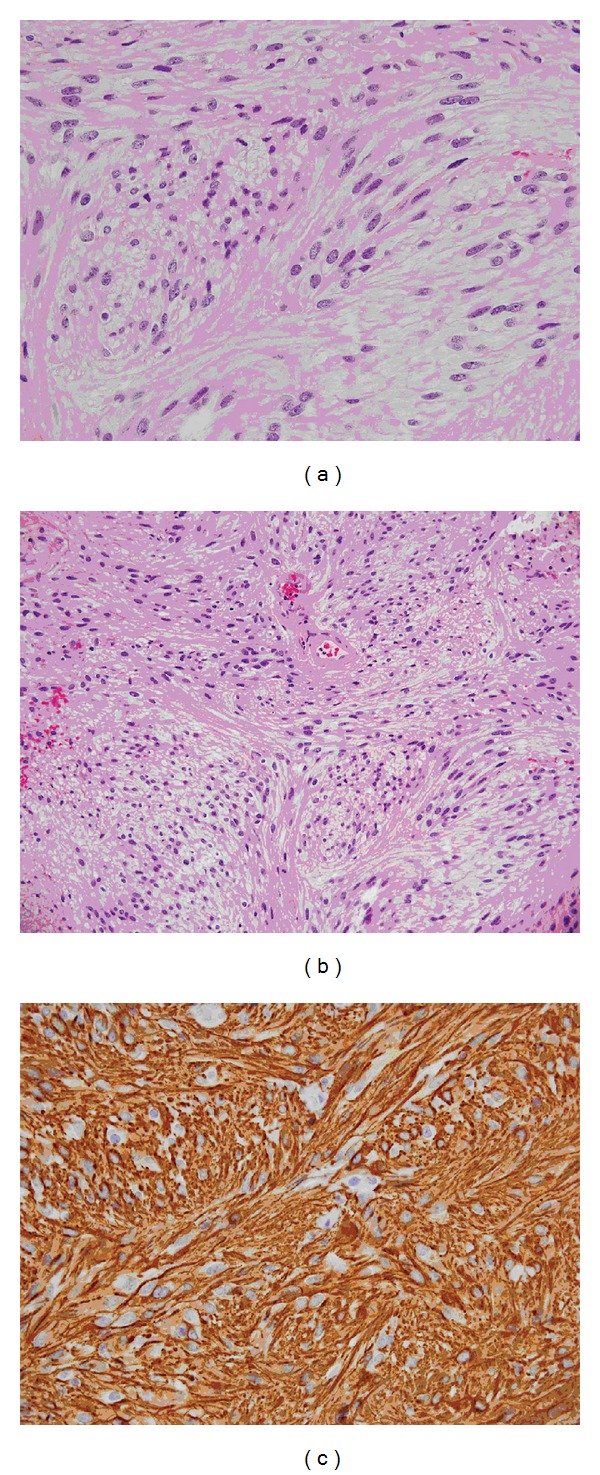
(a) Histological section showing spindle cell neoplasm of moderate cellularity arranged in interlacing fascicles. Hyalinized blood vessels and foci of hemosiderin pigment deposits are present. (b) Histological section showing neoplastic cells with oval to elongated nuclei with speckled chromatin and small nucleoli. (c) Histological section demonstrating cells that stained strongly positive for GFAP.

The frozen section showed spindle cells with oval and elongated nuclei with occasional hemosiderin deposits present (Figures 3(a) and 3(b)). There was moderate cellularity with no significant mitotic figures. A preliminary diagnosis of schwannoma was made based on the findings from the frozen section; however, immunohistochemical staining of the permanent section revealed strong immunoreactivity for glial fibrillary acidic protein (GFAP) with intermittent S-100 positivity, confirming that the tumor was of the tanycytic type of ependymoma (Figure 3(c)).

### 2.4. Postoperative Course

Because GTR of an ependymoma was achieved, no adjuvant therapy was implemented. The patient's postoperative course was uneventful. He experienced improvement in his bilateral lower extremity numbness with slight initial worsening in his upper extremity numbness, which later improved to better than baseline. The postoperative MR imaging showed complete resection with no evidence for recurrence. Sixteen months after surgery, the patient is free from recurrence.

## 3. Discussion

Tanycytic ependymoma is a rare form of ependymoma that was initially described by Friede and Pollak in 1978 [1]. Like its cellular counterpart, it is a WHO grade II tumor in histology and behavior [2]. Interestingly, however, it does not carry the typical histological features of the cellular form and often bears close resemblance to both schwannoma and pilocytic astrocytoma, making its diagnosis a conundrum [3].

The tumor cells are derived from the tanycyte, which is a bipolar cell with long processes within the neuropil, bridging the ependymal lining with the capillary wall. Given its peculiar intertwined position, it is thought to function in establishing communication between the cerebrospinal fluid, the brain parenchyma, and the vasculature [4]. Tanycytes most often arise in the vicinity of the lateral or fourth ventricle and the spinal cord. In the cerebrum, tanycytes are most commonly located along the lateral wall of the third ventricle. In the spinal cord, however, they surround the central canal and radiate towards the grey matter [5–7]. Published reports indicate that most adult ependymomas are located in the spinal cord in an extra- or intramedullary location [8]. The reported tanycytic forms of ependymoma are almost always intramedullary lesions. Excluding our case, 25 other cases of tanycytic ependymomas have been reported in the literature, of which 17 cases involved the spinal cord and only 3 were extramedullary [2]. Our case of a cervical intramedullary tanycytic ependymoma raises the total number of spinal cord tanycytic ependymomas reported to 18 and exclusive cervical spine involvement to 7 (Table 1).

Tanycytic ependymoma is of low-to-moderate cellularity characterized by a flow of elongated cells with nuclear pleomorphism. Mitotic figures are usually absent. It has inconspicuous features that are not typical of ependymoma. The distinctive perivascular pseudorosette pattern of ependymoma cells is infrequently encountered [9]. In a simple smear, the cells conglomerate in a cohesive cluster with their bipolar long processes coursing away from the vessel walls. Their nuclei are oval to spindle shaped with evident pleomorphism. The cell processes are not fluffy as with the cellular type of ependymoma [10].

Tanycytic ependymoma cells may mimic other tumor cells with similar features under light microscopy often lending to a challenging diagnosis. The spindle appearance of the cells, for instance, is similar to that of schwannoma cells. One distinguishing feature on a simple stain, however, is that tanycytic ependymoma cells are more uniform and their nuclei more oval compared with those of schwannoma cells. Similarly, the long processes arising from tanycytic ependymoma cells resemble those of pilocytic astrocytoma. Again, there are discerning features that set the tanycytic ependymoma cells apart from astrocytoma, such as the absence of Rosenthal fibers, the presence of large ovoid nuclei, tight perivascular packing of the cells, and their isomorphic cellular appearance [3, 9]. Those important subtleties can be missed when viewing under the light microscope with simple stains. In such instances, the pathologist should resort to immunohistochemical as well as ultrastructural characteristics that can help characterize the tumor type. Tanycytic ependymoma stains positive for GFAP and vimentin but rarely stains S-100. This helps to differentiate it from schwannoma, which is S-100 positive and GFAP negative. This histological predicament was well highlighted in our case, in which the intraoperative diagnosis on frozen section was thought to be schwannoma, but later immunohistochemical staining of the permanent section confirmed it was consistent with a tanycytic ependymoma. Although, pilocytic astrocytoma is GFAP positive, it almost never stains for vimentin, which helps to discriminate it from tanycytic ependymoma [11]. The electron microscopic features of well-developed cellular intermediate junctions as well as microvilli and cilia explain the ependymal nature of the tumor cells and narrow the likely diagnoses to schwannoma and ependymoma [2, 4, 9].

Radiographically, ependymoma usually enhances on T1-weighted imaging after administration of contrast agent and is often associated with a syrinx or hematoma. Whereas ependymoma is almost always intramedullary, schwannoma is almost always an extramedullary lesion that also avidly enhances on T1-weighted imaging with gadolinium contrast enhancement (Figures 1(b) and 1(c)). Pilocytic astrocytomas rarely enhance [11]. All the aforementioned histological and radiographic hallmarks are important in making a correct diagnosis, which has far-reaching implications with regards to anticipated prognosis, followup, and adjuvant therapy.

Intramedullary ependymomas of the cervical spine usually have an identifiable plane that allows for the safe achievement of GTR. Patients may experience initial neurological decline in the early postoperative period, and that risk increases with older patients. Fifty percent of patients who experience initial decline in their postoperative neurological status show an improvement to their preoperative baseline with continued improvement over the next year. Late improvement in motor, sensory, and bladder dysfunction may continue, especially in patients in whom a surgical plane could be identified [12–14]. With ependymomas, trends towards neurological improvement can be expected between 1 and 3 months and continue up to a year. Dysesthesias begin to improve within a month of surgery and are better by a year [13]. Regardless of tumor histopathology in patients with cervical intramedullary tumors, preoperative neurological status and GTR with an identifiable plane remain the best predictors of overall functional outcome [12].

## 4. Conclusion

 A rare case of spinal tanycytic ependymoma was presented in a 50-year-old-man. A careful and detailed histological inspection with utilization of immunohistochemical stains and ultrastructural microscopy may be necessary to distinguish tanycytic ependymoma from other neoplasm such as schwannoma and pilocytic ependymoma.

## Figures and Tables

**Table 1:** Demographic and clinical characteristics of  18 patients with spinal cord tanycytic ependymoma cases.

Author	Sex	Age (years)	Affected level	Location	Treatment	Prognosis
Friede and Pollak, 1978 [1]	F	38	C6-T3	Intradural	STR	Unknown
	F	46	T7-L2	Intradural	Unknown	Unknown
	M	45	C1-T1	Intradural	Unknown	Unknown
	M	17	C1-C3	Intradural	Unknown	Unknown
	F	36	C1-C6	Intradural	PR	Recurrence
	F	35	T10-T11	Unknown	Unknown	Unknown

Spaar et al., 1986 [15]	F	52	T6-T7	Intradural	GTR	Death due to post-operative complication

Langford and Barré, 1997 [4]	M	32	C	Intradural	GTR	No recurrence (2 yr)

Kawano et al., 2001 [9]	M	45	T3-T4	Intradural	GTR	No recurrence (9 yr)
	F	36	C3-C6	Intradural	Unknown	No recurrence (9 yr)
	F	55	C7-T2	Intradural	Unknown	No recurrence (2 yr)

Kobata et al., 2001 [16]	M	30	T6-T11	Extradural	STR	No regrowth (2 yr)

Ueki et al., 2001 [17]	F	13	C7-T2	Intradural	GTR	Unknown

Dvoracek and Kirby, 2001 [3]	F	31	L5-S1	Intradural	STR	No regrowth (8 months)

Boccardo et al., 2003 [18]	F	39	C5-C6	Intradural	GTR	No recurrence (2 yr)

Sato et al., 2005 [11]	M	58	C2-C4	Intradural	STR	No recurrence (2 yr)

Ishihama et al., 2011 [8]	F	40	T10-T11	Extradural	GTR	No recurrence (16 months)

This paper	M	50	C3-C5	Intradural	GTR	No recurrence (16 months)

GTR: gross total resection; STR: subtotal resection; PR: partial resection.

## References

[1] Friede RL, Pollak A (1978). The cytogenetic basis for classifying ependymomas. *Journal of Neuropathology and Experimental Neurology*.

[2] Kleihues P, Cavenee W (2000). *Pathology and Genetics of Tumours of the Nervous System. World Health Organization Classification of Tumours*.

[3] Dvoracek MA, Kirby PA (2001). Intraoperative diagnosis of tanycytic ependymoma: pitfalls and differential diagnosis. *Diagnostic Cytopathology*.

[4] Langford LA, Barré GM (1997). Tanycytic ependymoma. *Ultrastructural Pathology*.

[5] Lim BS, Park SQ, Chang UK, Kim MS (2010). Spinal cord tanycytic ependymoma associated with neurofibromatosis type 2. *Journal of Clinical Neuroscience*.

[6] Rafols JA, Goshgarian HG (1985). Spinal tanycytes in the adult rat: a correlative Golgi gold-toning study. *Anatomical Record*.

[7] Sara A, Bruner JM, Mackay B (1994). Ultrastructure of ependymoma. *Ultrastructural Pathology*.

[8] Ishihama H, Nakamura M, Funao H (2011). A rare case of spinal dumbbell tanycytic ependymoma. *Spine*.

[9] Kawano N, Yagishita S, Oka H (2001). Spinal tanycytic ependymomas. *Acta Neuropathologica*.

[10] Ng HK (1994). Cytologic features of ependymomas in smear preparations. *Acta Cytologica*.

[11] Sato K, Kubota T, Ishida M, Handa Y (2005). Spinal tanycytic ependymoma with hematomyelia—case report. *Neurologia Medico-Chirurgica*.

[12] Garcés-Ambrossi GL, McGirt MJ, Mehta VA (2009). Factors associated with progression-free survival and long-term neurological outcome after resection of intramedullary spinal cord tumors: analysis of 101 consecutive cases—clinical article. *Journal of Neurosurgery*.

[13] Hanbali F, Fourney DR, Marmor E (2002). Spinal cord ependymoma: radical surgical resection and outcome. *Neurosurgery*.

[14] Sandalcioglu IE, Gasser T, Asgari S (2005). Functional outcome after surgical treatment of intramedullary spinal cord tumors: experience with 78 patients. *Spinal Cord*.

[15] Spaar FW, Blech M, Ahyai A (1986). DNA-flow fluorescence—cytometry of ependymomas. Report on ten surgically removed tumours. *Acta Neuropathologica*.

[16] Kobata H, Kuroiwa T, Isono N, Nagasawa S, Ohta T, Tsutsumi A (2001). Tanycytic ependymoma in association with neurofibromatosis type 2. *Clinical Neuropathology*.

[17] Ueki K, Sasaki T, Ishida T, Kirino T (2001). Spinal tanycytic ependymoma associated with neurofibromatosis type 2—case report. *Neurologia Medico-Chirurgica*.

[18] Boccardo M, Telera S, Vitali A (2003). Tanycytic ependymoma of the spinal cord. Case report and review of the literature. *Neurochirurgie*.

